# β-Alanine supplementation and military performance

**DOI:** 10.1007/s00726-015-2051-9

**Published:** 2015-07-24

**Authors:** Jay R. Hoffman, Jeffrey R. Stout, Roger C. Harris, Daniel S. Moran

**Affiliations:** Institute of Exercise Physiology and Wellness, Sport and Exercise Science, University of Central Florida, Orlando, FL 32816 USA; Junipa Ltd, Newmarket, UK; School of Health Science, Ariel University, Ariel, Israel; Givat Washington College of Education, Givat Washington, Israel

**Keywords:** Soldiers, Performance, Dietary supplement, Nutrition

## Abstract

During sustained high-intensity military training or simulated combat exercises, significant decreases in physical performance measures are often seen. The use of dietary supplements is becoming increasingly popular among military personnel, with more than half of the US soldiers deployed or garrisoned reported to using dietary supplements. β-Alanine is a popular supplement used primarily by strength and power athletes to enhance performance, as well as training aimed at improving muscle growth, strength and power. However, there is limited research examining the efficacy of β-alanine in soldiers conducting operationally relevant tasks. The gains brought about by β-alanine use by selected competitive athletes appears to be relevant also for certain physiological demands common to military personnel during part of their training program. Medical and health personnel within the military are expected to extrapolate and implement relevant knowledge and doctrine from research performed on other population groups. The evidence supporting the use of β-alanine in competitive and recreational athletic populations suggests that similar benefits would also be observed among tactical athletes. However, recent studies in military personnel have provided direct evidence supporting the use of β-alanine supplementation for enhancing combat-specific performance. This appears to be most relevant for high-intensity activities lasting 60–300 s. Further, limited evidence has recently been presented suggesting that β-alanine supplementation may enhance cognitive function and promote resiliency during highly stressful situations.

## Introduction

During prolonged high-intensity military training or simulated combat exercises, significant decreases in physical and cognitive performance measures have been reported (Lieberman et al. 2002; Nindl et al. 2007; Weeks et al. 2010; Welsh et al. 2008). To compensate for the physiological and psychological fatigue associated with military training and combat, a number of pharmacological interventions have been examined. However, recently several members of the US Army Medical Corps have expressed a need to examine non-pharmacological alternatives to counteract the fatigue associated with military combat (Russo et al. 2008). The use of nutritional supplements among military personnel is quite common. Fifty-three percent of the American soldiers at various military installations around the world (outside of the combat theater) indicated that they used nutritional supplements on a regular basis (Lieberman et al. 2010). Knapick et al. (2014) estimated the prevalence of dietary supplement use for the US Army, Navy, Air Force and Marine Corps men to be 55, 60, 60 and 61 %, respectively; while for women the corresponding values were 65, 71, 76 and 71 %, respectively. Cassler et al. (2013) have indicated that up to 72 % of the US Marines deployed to Afghanistan used a nutritional supplement. However, little is known regarding the efficacy or safety of many of these supplements with regard to various specific military performances.

With regard to tactical performance, nutritional supplements can be divided into two separate categories: supplements to prepare personnel for the rigors of combat and supplements that are used for acute performance improvements. In addition, some supplements consumed during training are also intended to assist in recovery from training. Supplements that have been suggested to enhance physical development of military personnel should improve strength and muscle size during training. Often, these types of supplements are consumed in conjunction with strength and conditioning programs.

These supplements may also have an important role during sustained combat scenarios where high intensity and prolonged activity can often impair the physical capability of the operator (Committee on Dietary Supplement Use by Military Personnel, (2008). One of the more popular supplements being used by competitive athletes to improve athletic performance today is β-alanine (Blancquaert et al. 2015; Maughan et al. 2011). The efficacy of β-alanine to enhance athletic performance has been delineated in several recent reviews (Artioli et al. 2010; Derave et al. 2010; Hobson et al. 2012; Sale et al. 2010). However, a recent review on the efficacy of β-alanine in military performance suggested that the strength of evidence was not sufficient to support the use of β-alanine by military personnel (Ko et al. 2014). This review, however, was published prior to any studies investigating β-alanine supplementation of military personnel being conducted and, in addition, largely ignored the biochemical evidence underpinning its known physiological effects. Since Ko et al. (2014), two papers have been published examining the efficacy of β-alanine supplementation on military-specific tasks. As such, an updated review appears to be warranted. This review will examine the potential benefit of β-alanine supplementation among tactical personnel to help prepare them for the rigors of combat. It will also critically discuss investigations on β-alanine ingestion and performance in both competitive and tactical athletes, and provide physiological evidence for the use of β-alanine in the military population.

## β-Alanine supplementation

Β-Alanine is a non-proteogenic amino acid, and is one of the two constituents of carnosine (histidine being the second constituent). The combination of β-alanine raises the pKa of the imidazole ring of the histidine residue to 6.83, placing it in the center of the exercise intracellular pH transit range and enabling it to act as a highly effective pH buffer. The combination with β-alanine further renders the histidine moiety inert to proteogenic reaction with other amino acids, enabling high concentrations to be accumulated in muscle cells, further enhancing its quantitative role as a pH buffer. β-Alanine is considered to be the rate-limiting step in carnosine synthesis in at least muscle, due to its low rate of synthesis in vivo (Bauer and Sculz 1994; Harris et al. 2006). Thus, the aim of β-alanine supplementation is to increase the carnosine content in skeletal muscle and through this also the intracellular buffering capacity, enabling a greater tolerance of sustained anaerobic activity.

Carnosine is found in the skeletal muscle of all vertebrates (Harris et al. 1990), but is especially high in mammals that rely on anaerobic metabolism to fuel their activity. The relationship between carnosine content and muscle buffering capacity is strongly positive across all species (Harris et al. 1990; Abe 2000; Parkhouse et al. 1985), with a significantly greater concentration of carnosine found in type II compared to type I muscle fibers (Harris et al. 1998; Hill et al. 2007; Kendrick et al. 2009). Based upon its cross-species distribution, carnosine’s primary physiological role in muscle appears to be as a pH buffer (Abe 2000; Harris et al. 2006), moderating the decline in intracellular pH with lactate accumulation during intense exercise, or exercise performed under hypoxia. In humans, accumulating evidence suggests that high concentrations of carnosine within muscle delays the onset of peripheral fatigue with intense exercise lasting 1–5 min (Hobson et al. 2012). In only one study has high-intensity training alone been suggested to majorly increase muscle carnosine concentrations (Suzuki et al. 2004). Such an effect would require an increase in hepatic β-alanine synthesis and/or an increased rate of transport into muscle fibers. Suzuki et al. (2004) reported a doubling in the muscle carnosine content following 8 weeks of sprint training involving a total training time of 14 min (i.e., single 30-s sprints performed twice per week for the first 2 weeks and two 30-s sprints performed twice per week for the next 6 weeks with 20-min recovery between sprints). However, most other studies examining high-intensity training for durations ranging from 4 to 16 weeks, and where the total training time was in excess of that of Suzuki et al. (2004), have been unable to confirm these results (Kendrick et al. 2008, 2009; Mannion et al. 1994). An additional effect of training on the muscle carnosine content has recently been reported by Bex et al. (2014). In addition, this suggested that the effect of β-alanine supplementation may be increased with training, although a similar effect was not observed by Kendrick et al. (2009) when measured in different muscle fiber types.

Diet has a significant effect in humans on muscle carnosine concentrations. Muscle carnosine concentrations in omnivores are significantly higher than those seen in vegetarians (Everaert et al. 2011; Harris et al. 2007). In vegetarians, the only source of β-alanine for muscle synthesis is from uracil degradation in the liver. In humans, either because the process is slow or the availability of uracil for degradation is limited, in vitro β-alanine synthesis is sufficient only to support relatively low levels of carnosine synthesis in muscle. As a result, dietary intake of β-alanine through red meat, poultry and fish consumption is required if the muscle carnosine concentration is to be further increased (Harris et al. 2007). Ingestion of ~200 g of chicken breast meat, or 150 g of turkey breast meat, would result in an increase in the plasma bioavailability of β-alanine equivalent to an 800 mg β-alanine supplement (Harris et al. 2006). Considering that the daily dose range in supplementary β-alanine is from 1.6 to 6.4 g, this would require the consumption of between  ~400 and 1600 g of chicken breast or ~300–1200 g of turkey breast per day. Thus, the direct use of β-alanine as a supplement appears, today, to be the most effective means to increase dietary intake with the objective of elevating muscle carnosine concentrations.

## Kinetics of β-alanine ingestion

A 10 mg kg^−1^ dose of β-alanine (equivalent to an 800 mg dose for a 80 kg individual) will cause an increase of β-alanine in plasma that peaks between 30 and 40 min following ingestion (Harris et al. 2006). Its half-life (time at which there is a 50 % reduction of peak concentration) has been reported to be 25 min, while return to the baseline concentrations occurs 3 h post-ingestion (Harris et al. 2006). The use of sustained-release tablets that slow the rate of β-alanine release into circulation have been shown to increase retention with a reduction in the amount lost via urine. As a result, the percentage of β-alanine potentially available for carnosine synthesis in the muscle is increased (Harris et al. 2008). Decombaz et al. (2012) have reported that a one-time ingestion of a 1.6 g of slow-release β-alanine capsule in 11 healthy adults (6 males and 5 females) resulted in a smaller peak plasma concentration than a similar dose of a regular release capsule (82 vs. 248 μmol L^−1^, *p* < 0.001), a delayed time to peak concentration (1.0 vs. 0.5 h, *p* < 0.01), but no difference in plasma bioavailability when the area under the respective plasma concentration curves (AUC) was compared. With sustained-release tablets, the period over which the plasma β-alanine concentration is increased is prolonged, with the potential for a greater increase in muscle carnosine.

### Dose–response for β-alanine ingestion and muscle carnosine synthesis

It has been suggested that a daily dosing regimen of 1.6–6.4 g day^−1^ results in significant increases in muscle carnosine concentrations (Kendrick et al. 2008, 2009; Hoffman et al. 2012; Stellingwerff et al. 2012a). Doses higher than 6.4 g day^−1^ have generally not been examined due to the greater risk of symptoms of paresthesia occurring. However, most of these studies were conducted prior to the availability of a sustained-release formulation of β-alanine, which permits greater doses without the risk of paresthesia (Harris and Stellingwerff 2013). The increase in muscle carnosine content with β-alanine supplementation appears to be dependent upon several factors that include training history, dose and duration of use (see Table 1). Stellingwerff et al. (2012a) appear to be the only study to directly compare multiple dosing strategies and demonstrated that over a period of 4 weeks a higher dose (3.2 g day^−1^of β-alanine) resulted in a greater increase in carnosine content in both fast-twitch (gastrocnemius) and slow-twitch (tibialis anterior) muscle fibers than a lower dose (1.6 g day^−1^). Stellingwerff et al. (2012a) also reported that muscle carnosine levels are significantly increased with as little as 1.6 g day^−1^ following only 2 weeks of supplementation. Other studies have generally not compared different dosing schemes. Baguet et al. (2009) provided 4.8 g day^−1^ for 5–6 weeks and reported a 23 % increase in carnosine content of the gastrocnemius of recreationally trained men. In a subsequent study, the same research team investigating elite athletes ingesting a higher dose (5.0 g day^−1^) for a slightly longer duration (7-weeks) reported an increase in muscle carnosine content of 28 % in the gastrocnemius (Baguet et al. 2010). This study, however, used rowers who primarily rely on their upper body during training and competition. Derave and colleagues (2007) examining track and field athletes reported a 37 % increase in carnosine content in the gastrocnemius following 4.8 g day^−1^ for 4–5 weeks. Interestingly, Hoffman et al. (Hoffman et al. 2014) examining elite combat soldiers ingesting 6.4 g day^−1^ of sustained-release tablets for 4 weeks reported a 28 % increase in the carnosine content of the gastrocnemius muscle. Thus, doses ranging in amounts from 4.8 to 6.0 g day^−1^ for 4–7 weeks will result in an increase in carnosine content in the gastrocnemius ranging from 23 to 28 % in recreationally trained individuals, but possibly higher levels in competitive athletes, suggesting a potential a synergistic effect from training.

**Table 1:** Dose–response of β-alanine ingestion

Study	Participants	Study duration (weeks)	β-Alanine dose (g day^−1^)	Method of analysis	Muscle	Change in muscle carnosine			
Hill et al. (2007)	25 Recreationally trained males	10	Weeks 1–4 4.0–6.4 (800 mg ↑ per week) Weeks 5–10 6.4	Muscle biopsy	VL	16.5 ± 2.8 Type I 17.0 ± 2.6 Type II mmol kg^−1^dm			
Derave et al. (2007)	15 Track and field athletes	4–5	2.4–3.6 (week 1)	MRS	GAST	37 %			
			4.8 (week 2–4/5)		Soleus	47 %			
Kendrick et al. (2008)	26 Male physical education students	10	6.4	Muscle biopsy	VL	12.8 ± 8.0 mmol kg^−1^dm			
Baguet et al. (2009)	20 Recreationally trained males	5–6	4.8	MRS	GAST	23 %↑ (~1.8 mM)			
					TA	27 %↑ (~1.7 mM)			
					Soleus	39 %↑ (~2.2 mM)			
Kendrick et al. (2009)	14 Male physical education students	4	6.4	Muscle biopsy	VL	Trained leg 9.63 ± 3.92 (52.2 % ↑) Untrained leg 6.55 ± 2.36 (28.3 % ↑) mmol kg^−1^dm			
Baguet et al. (2010)	17 Male +1 female elite rowers	7	5.0	MRS	GAST	28 % ↑(~1.3 mM)			
					Soleus	45 % ↑(~1.4 mM)			
Stellingwerff et al. (2012b)	31 Males	8	High–low dosing	MRS	GAST TA	Low–low		High–low	
			Weeks 1–4 3.2 Weeks 5–8 1.6			35.5 ± 13.3 % ↑ 21.9 ± 14.4 % ↑		44.5 ± 12.5 % ↑ 30.3 ± 14.8 % ↑	
			Low–low dosing						
			Weeks 1–8 1.6						
del Favero et al. (2012)	18 Older (60–80 years) males and females	12	3.2	MRS	GAST	85.4 % ↑			
Bex et al. (2014)	35 Males (10 nonathletes and 25 athletes)	3.3	6.4 SR	MRS		NA	Cyc	Kay	SW
					GAST	25 %	67 %	20 %	48 %
					Soleus	40 %	87 %	39 %	62 %
					Deltoid	63 %	46 %	65 %	82 %
Hoffman et al. (2014)	18 Male soldiers	4	6.0 SR	MRS	GAST	28 %			

*MRS* magnetic resonance spectroscopy, *VL* vastus lateralis, *GAST* gastrocnemius, *TA* tibialis anterior, *SR* sustained release, *NA* nonathletes, *Cyc* cyclists, *Kay* kayakers, *SW* swimmers

The frequent reporting of values as % change, however, may be misleading as the same absolute increase in muscle carnosine will represent a considerably higher percentage increase in a vegetarian or someone on a low meat diet with a low initial muscle content, than in a high meat eater with a higher β-alanine intake. Furthermore, it is the absolute increase only from which the effect on performance can be judged. Over the exercise pH transit range (pH 7.1 down to 6.1), the absolute increase in proton (H^+^)-buffering capacity (ΔBC) is 29.4 % of the absolute increase in muscle carnosine. The percent is calculated using a transformation of the standard Henderson–Hassselbalch equation (Henderson 1908; Hasselbalch 1917) to estimate the percentage of carnosine with an additional proton, i.e., %[CarnosineH], at any pH (in this case at pH’s 6.1 and 7.1), and assuming a pKa of 6.83 for carnosine (Tanokura et al. 1976):

$$ \% \left[ {\text{CarnosineH}} \right] \, = { 1}00/\left( { 1 { } + {\text{ antilog}}_{ 10} ({\text{pH}} - {\text{pKa}})} \right), $$

$$ \Delta {\text{BC }} = \, \% \left[ {\text{CarnosineH}} \right]_{{{\text{pH 6}}. 1}} - \, \% \left[ {\text{CarnosineH}} \right]_{{{\text{pH 7}}. 1}} . $$

In a review of data from several studies, Stellingwerff et al. (2012b) concluded that the increase in muscle carnosine was linearly related to the total amount of beta-alanine given. However, this represents an empirical interpretation of collected data, rather than any kinetic model. On the other hand, data of Stellingwerff et al. (2012a) and Harris et al. (2006) suggest that the synthesis of muscle carnosine may follow zero-order kinetics with respect to the availability of β-alanine, while the decay in muscle carnosine back to the pre-supplementation level follows first-order kinetics (Harris et al. 2009; Stellingwerff et al. 2012b). Such a model (where synthesis is zero order with respect to β-alanine availability, and is opposed by a first-order decline back to the pre-supplementation state) predicts that a steady-state plateau will eventually be reached with any dose where the rate of synthesis in muscle matches the rate of decay. However, the increase above the pre-supplementation muscle carnosine content attained at plateau will be directly related to the β-alanine dose (i.e., the absolute increase with 6.4 g day^−1^ will be twice that with 3.2 g day^−1^) and not to the total dose given. As indicated, the absolute increase in carnosine will determine the benefit on performance and therefore higher doses may be needed to achieve peak or greater effects.

As previously noted, Bex et al. (2014) reported that a combination of training and β-alanine ingestion appeared to have a greater effect than β-alanine ingestion alone on increasing muscle carnosine content. In their study, Bex et al. showed greater increases in muscle carnosine in the exercised muscles of athletes compared to non-exercised muscle in non-athletes. Specifically, 23 days of β-alanine supplementation at 6.4 g day^−1^ was reported to increase carnosine content in the deltoid muscle by 82 % in swimmers, but only 63 % in non-athletes. However, such an effect was not seen by Kendrick et al. (2009) following 4 weeks of β-alanine supplementation (6.4 g day^−1^), in trained and untrained vastus lateralis muscle. Subjects served as their own controls (i.e., one leg was exercised, while the other remained sedentary) and analyses included measurement of carnosine in different fiber types. No significant effect of training on the increase in carnosine was observed in different fiber types, or at the level of whole muscle. These measures were obtained from direct chemical analysis of muscle biopsies, whereas data of Bex et al. (2014) was obtained using magnetic resonance spectroscopy (MRS). Increases in the muscle carnosine content assessed using muscle biopsy procedures generally appear to be larger in magnitude than the changes reported from MRS assessments (Stellingwerff et al. 2012a).

Most supplement studies examining β-alanine ingestion have used a fixed or constant dose. Hill et al. (2007) examining 25 physically active male subjects used a dosing protocol beginning with 4.0 g day^−1^ for the first week which was increased by 800 mg per week until week 4, and then maintaining 6.4 g day^−1^ for an additional 6 weeks. The dose administered over the first 4 weeks (initially 50 mg kg^−1^ day^−1^, increasing to 80 mg kg^−1^ day^−1^ by the 4th week) resulted in a 58.8 % increase in the muscle carnosine concentration, while an additional 21 % increase was seen by week 10 (Hill et al. 2007). Similarly, Kendrick et al. (2008) reported increases in muscle carnosine concentrations (53.5 %) in physically active physical education students following 10 weeks of supplementation using 6.4 g day^−1^ for the entire supplementation period. The dose used was similar to that in the Hill et al. (2007) study. Thus, increases in carnosine content appear to be rapid during the initial stages of ingestion, but the rate of carnosine elevation begins to slow as supplementation is continued. This is consistent with the kinetic model proposed earlier.

Large variability exists in the carnosine content of type II and type I fibers. Harris and colleagues (1998; Hill et al. 2007; Kendrick et al. 2009) indicated that the carnosine content of type II fibers can be up to two times higher than that found in type I. Such differences could possibly impact the potential of different muscles to increase their carnosine content with β-alanine supplementation, although both Hill et al. (2007) and Kendrick et al. (2009) observed equal increases in types I and II muscle fibers when judged on the absolute change. Differences between muscles in fiber composition, reflected in differences in pre-supplementation carnosine contents, will inevitably affect the apparent effect of supplementation when this is expressed in relative terms. Derave et al. (2007) provided 4.8 g day^−1^ for 4–5 weeks in track and field athletes and reported a 47 % increase in the carnosine content of the soleus muscle, a predominantly slow-twitch muscle, but only a 37 % increase in the gastrocnemius, a predominantly fast-twitch muscle. However, given that the pre-supplementation level in the gastrocnemius will be higher than that in soleus, the difference in the absolute change is in fact much more similar. Baguet et al. (2010) reported nearly a twofold difference in the magnitude of carnosine content elevation between the soleus and gastrocnemius in elite female rowers ingesting 5.0 g day^−1^ for 7 weeks. However, such differences in relative effect again appear to be largely the result of the initial content as determined by differences in fiber composition between the soleus and gastrocnemius.

Based upon a linear regression of data from several studies, Stellingwerff and colleagues (2012a) estimated that a total of ~230 g of β-alanine over several weeks (1.6–6.4 g day^−1^) would result in a ~50 % increase in muscle carnosine. This suggested a linear dependency (*R*^2^ = 0.921) between the increase in muscle carnosine content and the total amount of β-alanine consumed. This was shown to be independent of muscle type. In a meta-analysis published by Hobson et al. 2012), data analysis indicated that supplementation with a total ingestion of 179 g of β-alanine (the median dose across all studies) resulted in a median performance improvement of 2.85 % compared with a placebo. However, the ‘model’ proposed by Stellingwerff et al. (2012a) is, as already noted, largely empirical and not based on any consideration of the kinetics of carnosine synthesis and degradation.

### Carnosine washout

Cessation of β-alanine ingestion results in a gradual return of muscle carnosine concentrations to baseline levels. In one study, 20 young physically active males were divided into two groups that received either 4.8 g day^−1^ of β-alanine or placebo for 6 weeks. Three weeks after supplement cessation, the mean carnosine concentrations in three muscles were reported to have decreased by 31.8 % (Baguet et al. 2009). Following 9 weeks of β-alanine ingestion, muscle carnosine concentrations returned to baseline levels. Following supplementation with 6.4 g day^−1^ for 4 weeks, Harris et al. (2009) suggested an exponential rate of decay back to the pre-supplementation level with a half-life (*t*_1/2_) of 8.6 weeks. Stellingwerff et al. (2012b) have proposed that the apparent rate of muscle carnosine decrease is a function of the initial increase above the pre-supplementation level. Participants who were reported to be high responders (i.e., saw a greater accumulation of muscle carnosine content) required a greater washout time to return to baseline levels (~15 weeks) than participants that were reported to be low responders (~6 weeks). Stellingwerff et al. (2012a) demonstrated that a 1.6 or 3.2 g day^−1^ for 8 weeks in healthy, but untrained males increased absolute muscle carnosine content by an average of $2.0 1 {\text{ mmol}}\;{\text{kg}}^{ - 1}_{\text{ww}}$. This absolute change was equivalent to a relative ~30–45 % increase (depending upon fiber type) in muscle carnosine content. Cessation of β-alanine ingestion resulted in a slow washout time (~15–20 weeks) with a decay rate of approximately 2 % per week. This decay rate was slower than that reported by Baguet et al. (2009) and may have been related to the higher baseline levels. Such a dependency of the decay rate on the degree of elevation above baseline is consistent with the decay following first-order kinetics.

## Ergogenic properties of β-alanine supplementation

The ergogenic role of β-alanine is not the result of any direct actions of the amino acid itself, but is derived from its ability to combine with histidine within muscle tissue to form carnosine. However, the evidence supporting the ergogenic benefit of elevating muscle carnosine concentrations is strong and is supported by a vast number of studies on the importance of intracellular pH regulation during muscle contraction. In a meta-analysis study, Hobson et al. (2012) found that the greatest ergogenic potential for elevated carnosine concentrations occurs during high-intensity exercise lasting 60–240 s in duration. Overall, significant (*p* < 0.001) differences in performance were found between the β-alanine and the placebo groups. The analysis included 360 subjects (174 with β-alanine supplementation and 186 with placebo) from 15 published manuscripts. No significant benefit in β-alanine ingestion was noted in performance durations lasting <60 s compared to placebo ingestion.

In placebo-controlled studies, β-alanine supplementation has been consistent in demonstrating significant performance benefits in both recreational and competitive athletic populations performing high-intensity activity (Hill et al. 2007; Hoffman et al. 2006, 2008a, b; Kendrick et al. 2008; Stout et al. 2006, 2007). In a double-blind, placebo-controlled study, ingestion of 6.4 and 3.2 g day^−1^ of β-alanine (days 1–6 and days 7–28, respectively) in 12 untrained young men for 4 weeks (high dose was titrated to the low dose following the first week of ingestion) was shown to improve physical working capacity at fatigue threshold (PWC_FT_) by 14.5 % (*p* < 0.05) in the β-alanine group (Stout et al. 2006). This difference was significantly (*p* < 0.004) greater than the placebo group that showed no change in physical working capacity. Stout et al. (2007) conducted a follow-up study, recruiting 22 untrained college-aged women in another placebo-controlled, double-blind protocol. The active group was supplied with 3.2 and 6.4 g day^−1^ of β-alanine (days 1–7 and days 8–28, respectively) for 4-weeks. The investigators reported a 12.6 % (*p* < 0.001) improvement in PWC_FT_ and a 2.5 % (*p* < 0.05) increase in time to exhaustion during a graded exercise cycle ergometry test compared to that seen in the placebo-supplemented group.

Studies in trained, competitive athletes supplemented with β-alanine for similar durations have reported similar results. Derave et al. (2007) in a double-blind, placebo-controlled study reported that 4 weeks of β-alanine supplementation (4.8 g day^−1^) in 15 male 400-m sprinters was significantly (*p* < 0.05) able to delay fatigue in repeated bouts of isokinetic exercise (5 sets of 30 maximal voluntary knee extensions). A second double-blind, placebo-controlled study providing 4.8 g day^−1^ of β-alanine or placebo for 4 weeks to experienced, resistance-trained strength/power athletes was undertaken by Hoffman et al. (2008b). The difference in training volume (total number of repetitions performed in the squat exercise per workout), reported as the difference between workouts performed at week 0 and week 4 of the study, was significantly (*p* < 0.05) greater in the athletes using β-alanine (9.0 ± 4.1 repetitions) compared to the placebo (0.3 ± 7.8 repetitions). In addition, the average mean power output per repetition for each set was significantly (*p* < 0.05) higher in athletes supplementing with β-alanine than those given the placebo.

Hoffman et al. (2008a) provided 4.5 g day^−1^ of β-alanine for 4 weeks to college football players. Initial performance testing which occurred following 2 weeks of supplementation revealed no significant differences in sprint times or fatigue rates during repeated (total of three) shuttle runs (30–35 s per run with a 2-min rest between each sprint). However, a strong trend (*p* = 0.07) was noted in fatigue rate during a 60-s Wingate anaerobic power test. As supplementation continued, examination of the player’s resistance training logbooks showed further trends (*p* = 0.09) toward a higher (9.2 %) volume of training (load x repetitions) in those athletes supplementing with β-alanine compared to placebo. Although the inability to see any effect on repeated sprints of approximately 30–35 s appears to be consistent with the results of Hobson et al. (2012), it is also likely that the 2 week supplementation period (only 63 g of β-alanine ingested) in trained athletes may not have been sufficient to increase the muscle carnosine content by an amount producing a measurable change in performance. The trend toward an improved fatigue rate in a 60-s maximal intensity bout of exercise is consistent with the physiological role that β-alanine supplementation has on buffering capacity and high intensity exercise of 60 s duration or more (Hobson et al. 2012).

Studies examining longer supplementation periods of β-alanine during performance events lasting more than 60-s in duration have also reported significant benefits. Baguet and colleagues (2010) provided 5 g day^−1^ of β-alanine in elite, competitive rowers. Following 7 weeks of supplementation, athletes ingesting β-alanine were 2.8 ± 4.8 s faster during a 2,000-m rowing time trial performance than their pre-supplement times. The placebo group was 1.8 ± 6.8 s slower than their pre-supplement times. Although these differences were not statistically significant (*p* = 0.07), these results suggest a trend in an elite group of athletes that may have practical significance. Furthermore, intramuscular carnosine content in the experimental group was significantly (*p* < 0.05) higher by 45 and 28 % in the soleus and gastrocnemius, respectively, and the change in muscle carnosine content was significantly correlated (*r* = 0.498, *p* = 0.042) to performance improvement in the rowing time trial. Two other studies (Ducker et al. 2013; Hobson et al. 2013) have independently confirmed a positive effect of 4 weeks of β-alanine supplementation (~80 mg kg^−1^) on 2000-m rowing performance with similar gains in each case. Based on all three studies, the conclusion is that performance in this type of sustained high-intensity exercise lasting 6–7 min is improved by β-alanine-facilitated muscle carnosine increase.

High-intensity exercise performed immediately following a prolonged bout of endurance exercise may also benefit from β-alanine supplementation. In a double-blind, placebo-controlled study, Van Thienen et al. (2009) provided 17 trained cyclists with β-alanine in gradated doses from 2 to 4 g day^−1^ for 8 weeks. Performance testing included a varied intensity [50–90 % of maximal lactate steady state (MLSS)] 110-min cycle ergometer time trial followed by a 10-min time trial at 100 % of their MLSS, which was followed by a 30-s sprint. The nine athletes consuming β-alanine showed a significant 11.4 % (*p* < 0.0001) and 5.0 % (*p* < 0.005) improvement in both peak and mean power, respectively, during the 30-s sprint performance, which was higher than the group that consumed the placebo (*n* = 8). The authors concluded that β-alanine supplementation can significantly enhance sprint performance at the end of an exhaustive endurance exercise bout.

As previously noted (Hill et al. 2007), cycle ergometer exercise time at 110 % power max, with a predicted time to exhaustion (TTE) of 2.5 min, was increased by 11.8 % after 4 weeks of β-alanine supplementation, and 15.9 % after 10 weeks. The increase in TTE was correlated with the increase in the muscle carnosine content. An almost identical effect of 4 weeks of β-alanine supplementation on performance using the same exercise test was independently observed by Sale et al. (2011).

In a strictly anaerobic exercise environment, Sale et al. (2011) observed a 13.2 % increase in isometric exercise time at 45 % maximal voluntary contraction time where this was close to the holding time predicted by the Rohmert equation (Rohmert 1960; Ahlborg et al. 1972). In this case, the additional proton load with the increase in isometric exercise time, where loss of H^+^ from muscle is prevented by occlusion of the local blood supply, closely matched the estimated increase in muscle buffering capacity from the carnosine increase.

There have only been a limited number of studies examining the effects of β-alanine ingestion on aerobic endurance performance. Jordan et al. (2010) reported that following 4 weeks of β-alanine ingestion (6.0 g day^−1^), a delay in blood lactate accumulation was observed in participants who were not trained aerobically during the supplement period; a decrease in aerobic capacity was also noted. This is not surprising considering that the physiological role of carnosine in muscle does not provide a strong mechanism for enhancing endurance performance. Nevertheless, Smith et al. (2009) reported significant (*p* < 0.05) improvements in VO_2_peak, time to fatigue and total work performed during endurance performance in recreationally active males following 6 weeks of high-intensity interval training (5–7 sets of 2-min intervals at 90 % max power output, with 1-min rest between each interval) and β-alanine ingestion (6.0 g day^−1^ for 21 days followed by 3.0 g day^−1^ for another 21 days). Although improvements were noted in both the supplement and placebo groups in these measures following 3 weeks of training, only the β-alanine group showed significant (*p* < 0.05) aerobic improvements after the 6 weeks of training. These results were confirmed by a subsequent study by Walter et al. (2010) examining recreationally active women. Even though there is no direct physiological benefit of β-alanine supplementation for endurance and aerobic performance, the combination of anaerobic high-intensity intervals training and β-alanine is likely to improve the quality of the high-intensity sprints performed, indirectly affecting aerobic capacity and cardiovascular fitness.

## Additional physiological effects of β-alanine supplementation

Since carnosine is located in other tissues besides skeletal muscle, such as the brain, it may also have additional physiological roles. Several studies have suggested that carnosine may serve as a neuroprotector (Boldyrev et al. 2010). This is supported by evidence demonstrating carnosine’s biological role as an antioxidant, antiglycating and ion-chelating agent (Boldyrev et al. 2004; Hipkiss et al. 1998; Kohen et al. 1988; Trombley et al. 2000). To date, studies examining the role of β-alanine supplementation and oxidative stress have been limited. One study examined the effect of 28 days of β-alanine ingestion (4.8 g day^−1^) on markers of oxidative stress during a 40-min treadmill run in moderately trained young women (Smith et al. 2012). The results of the study failed to demonstrate any antioxidant benefit in the β-alanine group, as no differences were noted in the antioxidant measures between the treatment and placebo groups. A follow-up study by the same research group examined 4 weeks of β-alanine supplementation (4.8 g day^−1^ of sustained release) in recreationally trained men (Smith-Ryan et al. 2014). Following a 40-min run to induce oxidative stress, results indicated that β-alanine ingestion had no significant influence on reducing exercise-induced oxidative stress, but 95 % confidence intervals did suggest a reduction in lipid peroxidation. Considering the limited response of β-alanine supplementation on endurance exercise performance, future research on the potential antioxidant benefits of β-alanine supplementation should examine in athletes performing aerobic activity.

The benefits of carnosine elevation may not be seen in skeletal muscle alone. A study investigating a rodent model demonstrated that daily 22.5 mmol kg^−1^ feedings of β-alanine ingestion can increase carnosine concentrations in the cerebral cortex and hypothalamus (Murakami and Furuse 2010). The increase in brain carnosine concentration was also associated with an increase in brain-derived neurotrophic factor (BDNF) and a decrease in 5-hydroxyindoleacetic acid concentrations, a metabolite of serotonin. These biochemical changes also corresponded to an improved time in a maze containing anxiolytic compounds. The results of the study suggested that β-alanine ingestion may reduce anxiety during stressful activities.

A recent study examined the effect of β-alanine ingestion on post-traumatic stress disorder (PTSD)-like behavioral changes in rodents exposed to a predator-scent stress (PSS) (Hoffman et al. 2015b). The validity of this model has been demonstrated in several studies (Cohen et al. 2004, 2012), and has been shown to be an effective approach to determine the effects of various interventions on the behavioral response to stress (Cohen et al. 2012). Animals were provided with 100 mg of a β-alanine/glucomannan blend per kg of body mass in a powder form for 30 days. A total of 30 mg of powder (80:20 blend) was dissolved in 25 ml of water and provided daily. Following 30 days of β-alanine ingestion, the animals were exposed to PSS. Results indicated that β-alanine ingestion in animals exposed to PSS was effective in attenuating some of the behaviors associated with exposure to PSS (e.g., greater time spent in the open arms of a maze, greater number of open arm entries and a lower anxiety index). In addition, animals that were exposed, but supplemented with β-alanine, saw a 19 % lower startle response (*p* = 0.085) and a 15 % lower behavioral freezing response (*p* = 0.148) than animals exposed and fed a normal diet. Animals that were exposed to PSS and fed a normal diet were observed to be significantly less active in the elevated maze and had a greater anxiety level compared to animals that were either unexposed, or were exposed and supplemented with β-alanine. Rats that were supplemented with β-alanine experienced significant elevations in carnosine concentrations in the hippocampus, cortex, hypothalamus, amygdala and thalamus segments of the brain. Elevations in brain carnosine concentrations were also associated with maintaining BDNF expression in the hippocampus, and elevations in brain carnosine levels were inversely associated with anxiety index (*r*’s ranging from −0.471 to −0.550, *p*’s < 0.002) and positively associated with improved time spent in the open arms (*r*’s ranging from 0.453 to 0.521, *p*’s < 0.003). The protective effects associated with elevations in brain carnosine appear to be related to a protection of BDNF expression in the hippocampus. The data from this study appear to support a potential role of β-alanine as a dietary supplement for the treatment or prevention of PTSD. Considering that the prevalence of PTSD among returning US military veterans from the Iraq and Afghanistan combat zones are reported to range from 10 to 17 % (Sundin et al. 2010), the use of β-alanine may provide for a potential non-pharmacological intervention strategy that provides potential physiological and psychological resilience during combat operations.

It is important to note that the use of β-alanine to enhance resilience to stress is yet to be seen in a human model. However, existing evidence does suggest that if β-alanine can increase brain carnosine concentrations in humans, it may be able to maintain focus, alertness and cognitive function during highly fatiguing, high-intense activity.

## β-alanine supplementation and military performance

A recent review evaluating the efficacy of β-alanine supplementation for military personnel indicated that the evidence did not support the use of β-alanine supplementation for military populations (Ko et al. 2014). However, that review paper failed to discuss the relevance of the physiological effect of β-alanine specific to the physiological stresses of military training and combat operations, and ignored the benefits of this supplement during relatively short high-intensity activity. Furthermore, that review did not include any study or publication on military performance and β-alanine supplementation, as there were no published studies at that time. Considering the physiological role associated with elevations in muscle carnosine concentrations, and the known effects of the fatiguing nature of military training, it is possible that the use of β-alanine supplementation would likely provide benefits in a military population during operational-specific tasks. Since the publication of Ko et al. (2014), there have been two studies that have examined the ergogenic effects of β-alanine supplementation on military performance. In a double-blind study, Hoffman et al. (2014) investigated the effects of 4 weeks of β-alanine supplementation (6 g day^−1^) in 20 elite soldiers of an Israel Defense Forces (IDF) combat unit performing high-intensity military activity that required maintaining high levels of physical performance, focus, and decision making under fatiguing conditions. During the 4-week study period, all participants took part in advanced military training tasks that included combat skill development, physical work under pressure, navigational training, self-defense/hand-to-hand combat and conditioning. The investigation revealed that 4 weeks of β-alanine supplementation was effective in maintaining lower body power and psychomotor performance. Peak jump power, target engagement speed, and shooting accuracy were significantly improved for the β-alanine group in comparison with the placebo group (*p* < 0.034, *p* < 0.039, and *p* < 0.012, respectively), but did not appear to have any significant effects on cognitive function. Improvements in marksmanship and target engagement speed following exhausting exercise were thought to be related to potential elevations in muscle carnosine concentrations (this was not measured) that was able to minimize fatigue in the muscle fibers responsible for maintaining both shooting positions (standing and kneeling) and also to keep the weapon steady during target acquisition and marksmanship.

In a follow-up study, Hoffman et al. (2015a) again examined elite soldiers (*n* = 18) in an IDF combat unit. Soldiers were provided 6.0 g day^−1^ of β-alanine for 30 days, or a similar dose of placebo. During this study, changes in both muscle (gastrocnemius) and brain carnosine content was assessed by MRS. Following the 30-day supplement period, significant elevations in muscle carnosine were observed that were similar to those previously reported in athletes (Derave et al. 2007). No changes though were seen in brain carnosine levels. Significant performance improvements were reported in the 50-m casualty carry task, but no changes were noted in the 2.5-km run, 1-min sprint, or repeated sprints (5 × 30 m). However, a significant difference was seen in cognitive function between the soldiers ingesting β-alanine and placebo. Soldiers ingesting the supplement had a significantly (*p* = 0.022) greater number of correct answers on the 2-min serial subtraction test than soldiers that consumed the placebo. The difference in cognitive performance seen in this present study and the previous study in soldiers was suggested to be related to differences in assessment location. In a previous study, the serial subtraction test was performed in a quiet area, whereas in the latter study the test was performed in the shooting range while continuous live fire was being directed at targets. This required the soldiers to maintain their focus despite the loud noise from the active firing line. The level of anxiety brought about by loud noise and mathematical problem solving has been shown to significantly decrease cognitive performance in soldiers (Nibbeling et al. 2014). The results appear to suggest that 30 days of β-alanine ingestion may enhance cognitive function to a greater extent than placebo in a stressful environment.

The mechanism proposed to enhance β-alanine ingestion and cognitive function was thought to be an elevation in brain carnosine levels. However, the study by Hoffman et al. (2015b) was unable to detect any significant change in brain carnosine levels. This may be a function of the limitations of the available technology. The 3 tesla magnet used for the MRS assessment may have lacked the sensitivity to detect small changes in brain carnosine. In addition, it may also be speculated that carnosine elevations in the brains of humans may be rapidly hydrolyzed back to histidine and β-alanine by the enzyme carnosinase. A specific isoform of this enzyme has been previously detected in the brain (Jackson et al. 1991). Thus, as histidine concentrations begin to elevate, it may serve as a precursor for the increase in brain histamine concentrations. Elevated histamine concentrations have been shown to reduce anxiety (Endou et al. 2001). Other potential mechanisms may include the maintenance of BDNF expression, but without an elevation in brain carnosine levels this mechanism becomes less clear. Additional work is clearly needed to further our understanding of the effects of β-alanine ingestion on brain carnosine concentrations in humans.

Fatigue, common during sustained and highly intense tactical situations, may jeopardize judgment in differentiating friend from foe in a split second decision. In consideration of the physiological role that carnosine is reported to have in the brain (e.g., potential anti-anxiety effects), β-alanine ingestion may increase cognitive function during periods of exhaustive activity. Thus, additional research is warranted examining the potential efficacy of β-alanine supplementation as a potential adjuvant in minimizing fatigue during military operations.

## Safety

The only side effect reported with β-alanine supplementation is paresthesia (Harris et al. 2006). Paresthesia is a sensation of numbing or tingling in the skin. Investigations reporting potential side effects from prolonged (greater than 15 weeks) supplementation protocols have not been seen. However, considering that β-alanine is a naturally occurring amino acid with an important physiological role in the body, it is likely a very safe supplement to use. Nevertheless, the long-term effect of β-alanine supplementation and the combinations with other supplements are unknown. Further studies are needed to better understand the benefits and safety of β-alanine along with the effects of different variables such as sex, age, stress, and physical fitness under various conditions such as climate, altitude, and nutrition.

Paresthesia has been commonly experienced when consuming β-alanine (>800 mg kg^−1^) in a non-sustained-release form (Harris et al 2006). Reports of paresthesia have not been reported in studies that use sustained-release formulations. Symptoms of paresthesia generally disappear within 60–90 min following supplementation (Stellingwerff et al. 2012b). Recently, two different studies were conducted on the mechanisms behind β-alanine-induced paresthesia. Liu et al. (2012) examined intradermal injections of β-alanine in mice, while MacPhee et al. (2013) examined orally ingested 3-g dose of β-alanine in humans. Both studies concluded that the skin sensations aroused by β-alanine are facilitated by Mas-related G-protein-coupled receptor D (MrgprD), which is expressed in the cutaneous sensory neurons. Although paresthesia appearance after 800 mg of β-alanine consumption does not seem to be a serious reaction, the symptoms under different military scenarios should be investigated (Ko et al. 2014).

## Summary

When β-alanine is ingested, whether through food or as a dietary supplement, it combines with histidine to form carnosine. Physiologically, elevations in muscle carnosine will increase intracellular buffering capacity. The efficacy of β-alanine supplementation has been supported through several studies examining sustained, high-intensity exercise in competitive and recreational athletes. Presently, only limited evidence exists that supports β-alanine supplementation for enhancing combat performance in soldiers under fatiguing conditions. Based on current research, β-alanine supplementation appears to be efficacious during high-intensity activity lasting 60–360 s. In addition, evidence indicating potential psychological benefits including enhanced cognitive function during stressful conditions and improved resiliency to stress from β-alanine ingestion should stimulate further inquiry into this area. Results of the investigations conducted on both competitive and tactical (i.e., soldiers) athletes provides strong evidence that supplementing with β-alanine will increase muscle carnosine level. Increases in muscle carnosine will improve performance during sustained, high-intensity activity and would likely benefit soldier performance during training and in specific combat scenarios and sustained operations. The role that elevations in carnosine may have in cognitive function needs further study.
